# The ApoB/ApoA-I ratio and the risk of in-stent restenosis following drug-eluting stent implantation in patients with coronary heart disease: a retrospective cohort study

**DOI:** 10.3389/fcvm.2026.1869582

**Published:** 2026-08-12

**Authors:** Yuang Cheng, Jixin Hou, Licheng Jiang, Qiulin Wang, Xinquan Wang, Jun Hu, Lu Li, Sen Liu, Mingyang Li, Peng Yan, Peng Zhou, Peijian Wang, Changqiang Yang

**Affiliations:** 1 School of Clinical Medicine, Chengdu Medical College, Chengdu, Sichuan, China; 2 Department of Cardiology, School of Clinical Medicine, The First Affiliated Hospital of Chengdu Medical College, Chengdu, Sichuan, China; 3 Key Laboratory of Aging and Vascular Homeostasis at Chengdu Medical College of Sichuan Province, The First Affiliated Hospital of Chengdu Medical College, Chengdu, Sichuan, China

**Keywords:** ApoB/ApoA-I ratio, cohort study, coronary heart disease, drug-eluting stent, in-stent restenosis

## Abstract

**Objective:**

The Apolipoprotein B/Apolipoprotein A-I (ApoB/ApoA-I) ratio has been strongly associated with the risk of cardiovascular disease. However, its association with in-stent restenosis (ISR) after drug-eluting stent (DES) implantation in patients with coronary heart disease (CHD) remains unclear.

**Methods:**

This retrospective study enrolled CHD patients who underwent follow-up coronary angiography 6–24 months after successful DES-based percutaneous coronary intervention (PCI). The ApoB/ApoA-I ratio was calculated, and participants were grouped by quartiles of the ratio. Multivariable logistic regression and restricted spline cubic (RCS) models assessed the association between the ApoB/ApoA-I ratio and ISR risk. Subgroup and sensitivity analyses were also conducted.

**Results:**

A total of 1,004 CHD patients (mean age 67.32 ± 10.63 years, 63.65% male) were included. 176 (17.5%) patients developed ISR over a median 12-month follow-up. The prevalence of ISR rose stepwise with increasing quartiles of the ApoB/ApoA-I ratio (5.6% vs. 11.2% vs. 20.3% vs. 32.6%, *P* < 0.001), and the ratio was higher in the ISR group than in the non-ISR group (0.86 ± 0.28 vs. 0.67 ± 0.21, *P* < 0.001). After full adjustment for confounders, the ApoB/ApoA-I ratio remained positively associated with ISR risk [per 1-SD increase: odds ratios (OR) = 2.39, 95% CI: 1.81–3.15, *P* < 0.001]. Using the first quartile as reference, the OR for the third and fourth quartiles were 3.48 (95% CI: 1.67–7.22, *P* = 0.001) and 6.09 (95% CI: 2.66–13.93, *P* < 0.001), respectively. Sensitivity analyses supported these findings. RCS analysis indicated a linear dose-response relationship between the ApoB/ApoA-I ratio and ISR risk (*P* for overall <0.001, *P* for nonlinear = 0.332). Subgroup analyses confirmed the consistent association across all subgroups (all *P* for interaction >0.05). Moreover, adding ApoB/ApoA-I ratio to the predictive model for ISR could contribute to an increase in C-statistics (0.732 vs. 0.768, *P* = 0.001), categorical net reclassifcation improvement (0.128, *P* = 0.001), and integrated discrimination improvement (0.053, *P* < 0.001).

**Conclusions:**

An elevated ApoB/ApoA-I ratio was significantly associated with an increased risk for ISR after DES-based PCI in CHD patients. Our findings suggested that the ApoB/ApoA-I ratio might serve as a useful predictor for ISR risk in this population.

## 1 Introduction

Coronary heart disease (CHD) remains a leading cause of death and disability worldwide, imposing a substantial burden on global healthcare systems (1). Since its introduction in the 1970s (2), percutaneous coronary intervention (PCI) has become a cornerstone in the management of this disease, evolving through multiple technological milestones-from balloon angioplasty to bare-metal stents (BMS), and subsequently to drug-eluting stents (DES) (3). Although DES significantly reduces in-stent restenosis (ISR) rates compared with BMS (4), ISR after DES implantation remains a persistent clinical challenge, with reported incidence ranging from 3% to 20% (5). ISR compromises long-term vessel patency and is associated with an increased risk of major adverse cardiovascular events, including myocardial infarction, repeat revascularization, and increased mortality (6, 7). While coronary angiography is considered as the gold standard for ISR diagnosis (8), its invasive nature and associated costs often lead to poor patient adherence to follow-up evaluations (9). This reluctance may lead to delayed ISR detection, thereby increasing the risk of subsequent adverse cardiovascular outcomes (10, 11). Moreover, the predisposing factors and underlying mechanisms of ISR are heterogeneous, rendering its management clinically challenging (8). Consequently, identifying a simple, reliable biomarker for the early detection of high ISR risk during post-PCI follow-up can facilitate timely intervention and improve outcomes, holding considerable clinical significance.

Dyslipidemia-driven neoatherosclerosis has been increasingly recognized as a key biological mechanism contributing to ISR (12, 13). However, traditional lipid biomarkers often lack precision in assessing atherogenic dyslipidemia. Apolipoprotein B (ApoB) is an insoluble structural protein that serves as the primary component of all atherogenic lipoproteins, including very-low-density lipoprotein (VLDL), intermediate-density lipoprotein (IDL), low-density lipoprotein (LDL), and chylomicrons (14). Because each such particle contains exactly one ApoB molecule, ApoB concentration provides a direct and accurate measure of the total number of atherogenic particles—offering a more precise assessment of cardiovascular risk than conventional parameters such as LDL cholesterol or non high-density-lipoprotein (HDL) cholesterol (15). In contrast, Apolipoprotein A-I (ApoA-I) is the principal structural and functional protein of HDL, playing a central role in reverse cholesterol transport and cellular cholesterol homeostasis (14). ApoA-I exerts potent anti-atherosclerotic effects (16) and is increasingly regarded as a superior alternative to HDL cholesterol for evaluating antiatherogenic capacity (17). Given these opposing roles, the ApoB/ApoA-I ratio has emerged as a robust indicator reflecting the dynamic balance between proatherogenic and antiatherogenic lipoproteins in plasma (18).

Given this pathophysiological rationale, the ApoB/ApoA-I ratio has been investigated extensively in cardiovascular risk prediction. Accumulating evidence supports the ApoB/ApoA-I ratio as a strong and independent predictor of atherosclerotic cardiovascular disease (19) and major adverse cardiovascular events (20). Although its association with coronary heart disease is well established (21), the specific relationship between the ApoB/ApoA-I ratio and DES-related ISR has not been elucidated. To date, no dedicated studies have investigated this association. To address this critical knowledge gap, we conducted the present study to evaluate the relationship between the ApoB/ApoA-I ratio and the risk of ISR in patients with coronary heart disease following successful DES-based PCI.

## 2 Materials and methods

### 2.1 Study population and design

**Figure 1:**
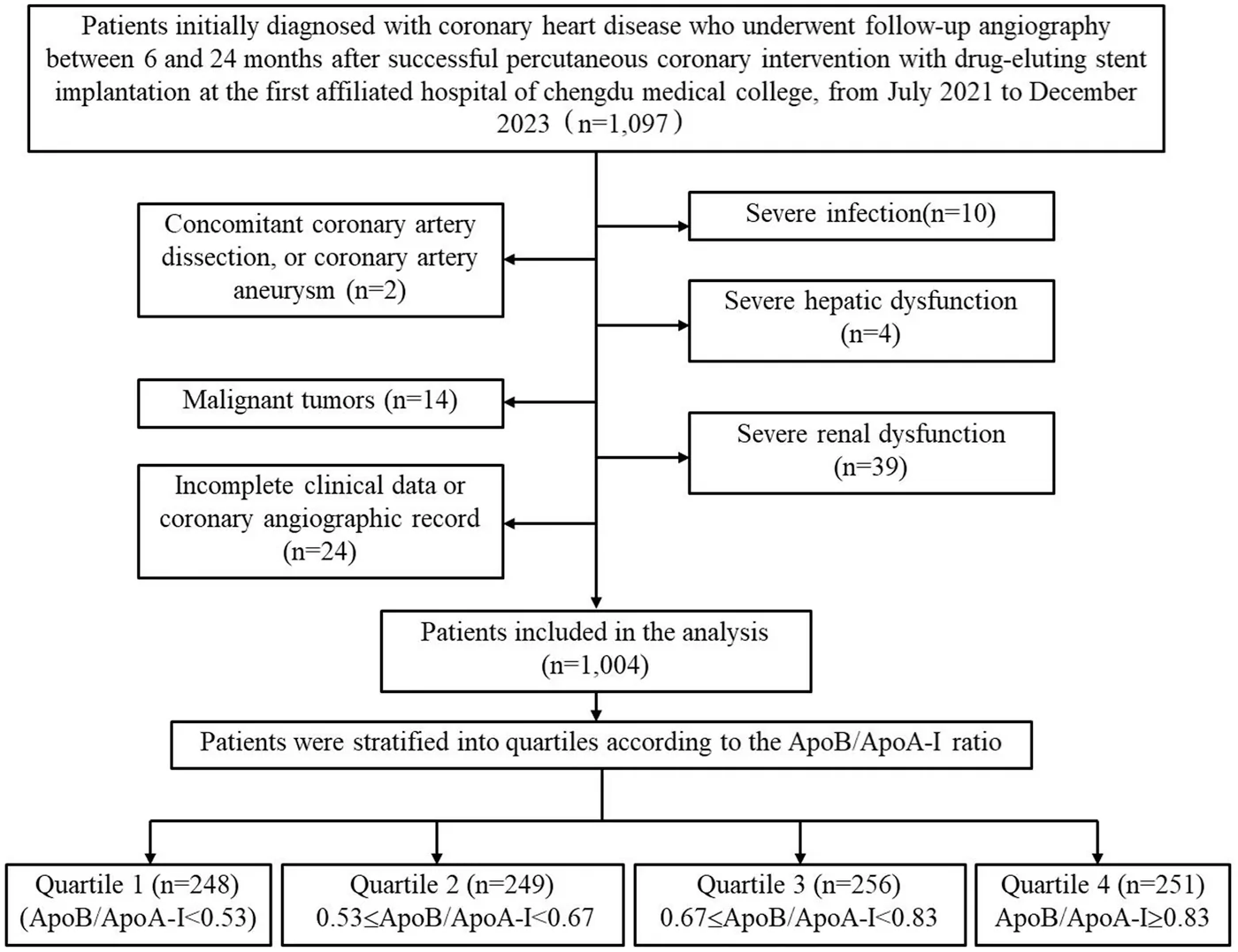
Flowchart of study participants.

A total of 1,097 patients who were initially diagnosed with CHD and underwent follow-up angiography ranging from 6 to 24 months after successful PCI with the implantation of at least one drug-eluting stent at the First Affiliated Hospital of Chengdu Medical College between July 2021 and December 2023 were reviewed retrospectively. All coronary angiography and PCI were performed in accordance with standard procedures and relevant guideline (22). Patients received a loading dose of aspirin and clopidogrel prior to the intervention, unless they were already on antiplatelet therapy. The choice of procedural strategy, stenting technique, and the use of glycoprotein IIb/IIIa inhibitors or intravascular ultrasound was determined by the operating surgeon. Patients were treated with standard dual antiplatelet therapy after PCI for at least one year. The main exclusion criteria were as follows: (1) severe hepatic insufficiency (*n* = 4); (2) severe renal dysfunction (*n* = 39); (3) concomitant coronary artery dissection or coronary artery aneurysm (*n* = 2); (4) severe infection (*n* = 10); (5) malignant tumors (*n* = 14); and (6) incomplete clinical data or coronary angiographic records (*n* = 24). Ultimately, 1,004 patients were enrolled in the final analysis. The detailed study flowchart was present in Figure 1. The study protocol was conducted in accordance with the Declaration of Helsinki and was approved by the Ethics Committee of the First Affiliated Hospital of Chengdu Medical College (approval number: 2025CYFYIRB-BA-068). Due to the retrospective design, the Ethics Committee waived the requirement for written informed consent.

### 2.2 Data collection and definitions

Clinical data, including demographics [sex, age, body mass index (BMI), smoking and drinking status], medical history, and laboratory tests, were extracted from the electronic medical record system. Laboratory analyses comprised hemoglobin, platelet (PLT), white blood cell (WBC), alanine aminotransferase (ALT), aspartate aminotransferase (AST), albumin, glucose, uric acid (UA), creatinine, estimated glomerular filtration rate (eGFR), brain natriuretic peptide (BNP) and serum lipid profiles, including triglyceride (TG), total cholesterol (TC), low-density lipoprotein cholesterol (LDL-C), high-density lipoprotein cholesterol (HDL-C), lipoprotein(a) [Lp(a)], Apolipoprotein A-I (ApoA-I) and Apolipoprotein B (ApoB). All assays were performed in the central laboratory of the First Affiliated Hospital of Chengdu Medical College. Blood samples were collected after patient admission to the hospital ward and before coronary angiography. Angiographic data were retrieved from cardiac catheterization laboratory records by trained physicians who were blinded to the study objective. Acute myocardial infraction (AMI) was defined according to the current European Society of Cardiology guidelines (23). Diabetes mellitus (DM) was defined as a prior diagnosis of diabetes, fasting blood glucose ≥7.0 mmol/L, HbA1c ≥6.5%, or use of glucose-lowering medication. Hypertension was defined as a history of hypertension with anti-hypertensive drugs or repeated blood pressure measurements ≥140/90 mmHg. The estimated Glomerular Filtration Rate (GFR) was calculated using a modified version of the Modification of Diet in Renal Disease (MDRD) equation adapted for the Chinese population (24). Body mass index (BMI) was calculated as weight (kg) divided by height squared (m^2^).

### 2.3 Follow-up angiography and evaluation of ISR

The clinical follow-up was conducted by trained personnel who were blinded to the study details, either via telephone or in-person clinic visits. All patients included in the analysis underwent follow-up angiography using the standard Judkins technique at 6–24 months after successful PCI. Angiographic assessments were performed by two independent, experienced cardiologists who were blinded to the study protocol, with any discrepancies adjudicated by a third senior cardiologist. ISR was defined as the presence of significant diameter stenosis (≥50%) within the stent segment or involving its 5-mm edges, as confirmed by angiography (8).

### 2.4 Statistical analysis

Continuous variables were presented as mean ± SD or median (interquartile range, IQR), and categorical variables as frequencies (n) and percentages (%). Group comparisons were performed using one-way analyses of variance (ANOVA) or Student's *t*-test for normally distributed variables, and the Kruskal–Wallis or Mann–Whitney *U* test for non-normally distributed variables; categorical variables were compared with the chi-square test or Fisher's exact test, as appropriate. To evaluate the association between the ApoB/ApoA-I ratio and ISR, multivariable logistic regression analyses were conducted with the ratio entered as both a continuous and a categorical variable. Four models were constructed: Model 1 (unadjusted); Model 2 (adjusted for age, sex, smoking, drinking, and BMI); Model 3 [further adjusted for DM, hypertension, AMI, WBC, PLT, glucose, UA, eGFR, TC, HDL-C, TG, BNP, and Angiotensin-Converting Enzyme Inhibitors (ACEI)/Angiotensin II Receptor Blockers (ARB) based on Model 2]; and Model 4 (additionally adjusted for the number of diseased vessels, number of implanted stents, and average stent diameter based on Model 3). Results were expressed as odds ratios (OR) with 95% confidence intervals (CI). Multicollinearity was assessed using variance inflation factors (VIF), with a VIF > 5 considered indicative of collinearity (see Supplementary Table S1). A sensitivity analysis was performed by excluding patients with acute myocardial infarction. To explore potential nonlinearity in the relationship between the ApoB/ApoA-I ratio and ISR risk, restricted cubic spline (RCS) analysis with five knots was applied, using the covariate adjustments from Model 4. Subgroup analyses were conducted to assess the consistency of the association across strata defined by sex, age (<65 vs. ≥65 years old), BMI (<24 vs. ≥24 kg/m^2^), eGFR (<90 vs. ≥90 ml/min/1.73m^2^), smoking, drinking, hypertension, and DM. Interaction terms were tested in these subgroups. The predictive performance of the ApoB/ApoA-I ratio for ISR was evaluated using receiver operating characteristic (ROC) curve analysis, from which the area under the curve (AUC) and the optimal cut-off value were determined. To assess whether the ApoB/ApoA-I ratio provided incremental predictive value beyond established risk factors, the C-statistics was calculated and compared using DeLong's test. Additionally, the categorical net reclassification improvement (NRI) and integrated discrimination improvement (IDI) were computed to further quantify the incremental predictive value of this ratio. All statistical analyses were performed using IBM SPSS Statistics (version 26.0), R software (version 4.3.3), and MedCalc 19.1 (MedCalc Software, Belgium). A two-tailed *P* < 0.05 was considered statistically significant.

## 3 Results

### 3.1 Baseline characteristics

Table 1 presented the baseline characteristics of the study participants. A total of 1,004 patients with coronary heart disease who underwent DES-PCI were enrolled. The mean age of the study population was 67.32 ± 10.63 years old, and 639 patients (63.65%) were male. Patients were stratified into four groups based on quartiles of the ApoB/ApoA-I ratio. Compared with those in the lower quartiles, patients in the higher ApoB/ApoA-I ratio quartiles had a higher proportion of male, smoking, drinking and AMI. They also exhibited higher values for the number of diseased vessels, number of implanted stents, BMI, hemoglobin, PLT, WBC, ALT, AST, eGFR, UA, TC, LDL-C, TG, along with lower levels of HDL-C and age. Furthermore, differences between the ISR (*n* = 176) and non-ISR (*n* = 828) group were analyzed (see Supplementary Table S2). Patients in the ISR group had a higher prevalence of smoking, drinking, use of ACEI/ARB, hypoglycemic agents and diabetes mellitus (DM). They also demonstrated higher levels of BMI, number of diseased vessels, number of implanted stents, total stent length, PLT, WBC, creatinine, UA, ApoB, ApoB/ApoA-I, TC, LDL-C, TG, and BNP.

**Table 1:** Baseline characteristics according to the quartiles of the ApoB/ApoA-I ratio.

Variable	Total (*n* = 1,004)	Quartile of ApoB/ApoA-I ratio				*P* value
		Q1 (*n* = 248) ApoB/ApoA-I < 0.53	Q2 (*n* = 249) 0.53 ≤ ApoB/ApoA-I < 0.67	Q3 (*n* = 256) 0.67 ≤ ApoB/ApoA-I < 0.83	Q4 (*n* = 251) ApoB/ApoA-I ≥ 0.83	
Age, years	67.32 ± 10.63	70.22 ± 8.50	68.71 ± 9.71	66.65 ± 10.74	63.78 ± 12.13	<0.001
Male, *n* (%)	639 (63.65)	123 (49.60)	162 (65.06)	171 (66.80)	183 (72.91)	<0.001
Smoking, *n* (%)	422 (42.03)	76 (30.65)	91 (36.54)	123 (48.04)	132 (52.59)	<0.001
Drinking, *n* (%)	239 (23.80)	45 (18.14)	56 (22.49)	64 (25.00)	74 (29.48)	<0.001
BMI, kg/m^2^	24.45 ± 3.24	23.75 ± 3.16	23.96 ± 3.02	24.78 ± 3.26	25.30 ± 3.29	<0.001
Number of diseased vessels	2.04 ± 0.89	1.86 ± 0.85	2.01 ± 0.87	2.11 ± 0.92	2.20 ± 0.90	<0.001
Number of implanted stents/patients	2.14 ± 1.41	1.95 ± 1.23	2.08 ± 1.33	2.20 ± 1.54	2.33 ± 1.49	0.018
Average stent diameter, mm/patients	3.10 ± 0.39	3.10 ± 0.39	3.12 ± 0.38	3.10 ± 0.39	3.10 ± 0.39	0.954
Total stent length, mm/patients	44.0 (25.13,69.00)	38.00 (25.25,61.75)	42.00 (24.00,69.00)	44.00 (24.00,70.50)	50.00 (28.00,77.00)	0.090
Hemoglobin, g/L	133.35 ± 18.73	128.51 ± 17.95	133.42 ± 18.52	134.70 ± 18.74	136.69 ± 18.81	<0.001
PLT, 10^9/L	183.23 ± 66.79	164.29 ± 57.95	183.79 ± 71.88	186.00 ± 61.41	198.56 ± 70.76	<0.001
WBC, 10^9/L	7.64 ± 3.17	6.59 ± 2.31	7.53 ± 3.29	7.93 ± 3.17	8.48 ± 3.49	<0.001
Albumin, g/L	41.17 ± 4.60	41.32 ± 4.37	41.54 ± 3.68	40.80 ± 4.19	41.04 ± 5.87	0.289
ALT, U/L	26.0 (18.00, 39.00)	23.00 (16.00, 33.00)	25.00 (18.00, 37.50)	26.00 (18.00, 40.00)	30.00 (20.00, 45.00)	<0.001
AST, U/L	27.0 (20.00, 42.00)	25.00 (19.00, 35.00)	26.00 (30.00, 37.00)	28.00 (20.00, 47.00)	29.00 (21.00, 73.00)	0.006
Creatinine, umol/L	74.00 (62.93, 88.70)	72.30 (61.13, 87.35)	73.3 (62.85, 88.25)	76.60 (64.25, 90.10)	73.80 (63.70, 87.60)	0.451
eGFR, ml/min/1.73 m^2^	96.64 (78.19, 115.38)	92.61 (75.31, 112.33)	96.82 (79.69, 114.70)	96.24 (77.59, 112.28)	101.14 (81.84, 120.22)	0.093
UA, umol/L	367.03 ± 99.88	350.73 ± 92.71	361.85 ± 94.71	377.48 ± 109.31	377.62 ± 99.64	0.005
Glucose, mmol/L	6.81 (5.57, 9.04)	6.62 (5.36, 8.67)	6.61 (5.48, 8.99)	6.96 (5.74, 9.14)	7.07 (5.59, 9.82)	0.073
ApoA-I, g/L	1.38 ± 0.30	1.59 ± 0.28	1.47 ± 0.27	1.34 ± 0.22	1.13 ± 0.22	<0.001
ApoB, g/L	0.93 ± 0.23	0.71 ± 0.16	0.88 ± 0.16	0.98 ± 0.17	1.13 ± 0.20	<0.001
ApoB/ApoA-I	0.70 ± 0.23	0.45 ± 0.06	0.60 ± 0.04	0.74 ± 0.4	1.01 ± 0.18	<0.001
TC, mmol/L	4.65 ± 1.14	3.99 ± 0.94	4.53 ± 1.01	4.76 ± 1.05	5.32 ± 1.14	<0.001
HDL-C, mmol/L	1.25 ± 0.35	1.44 ± 0.39	1.31 ± 0.34	1.17 ± 0.28	1.07 ± 0.24	<0.001
LDL-C, mmol/L	2.81 ± 0.97	2.04 ± 0.66	2.68 ± 0.74	2.99 ± 0.83	3.53 ± 0.97	<0.001
Lp (a), mg/L	198.00 (111.47, 377.40)	184.85 (112.25, 399.93)	212.30 (117.50, 410.80)	198.00 (113.00, 366.28)	195.00 (102.00, 345.00)	0.639
TG, mmol/L	1.64 (1.09, 2.52)	1.25 (0.90, 1.91)	1.56 (0.97, 2.24)	1.78 (1.24, 2.63)	2.01 (1.35, 3.10)	<0.001
BNP, ng/L	55.00 (22.00, 149.00)	59.00 (25.00, 148.00)	49.00 (20.00, 136.50)	52.00 (20.25, 148.50)	68.00 (25.00, 164.00)	0.387
DM, *n* (%)	294 (29.28)	66 (26.61)	73 (29.32)	73 (28.52)	82 (32.67)	0.509
Hypertension, *n* (%)	603 (60.06)	166 (66.94)	150 (60.24)	150 (58.59)	137 (54.58)	0.041
AMI, *n* (%)	283 (28.19)	37 (14.92)	63 (25.30)	82 (32.03)	101 (40.24)	<0.001
Postoperative medication	—	—	—	–	–	–
Aspirin	1,004 (100)	248 (100)	249 (100)	256 (100)	251 (100)	1.000
Clopidogrel/Ticagrelor	1,004 (100)	248 (100)	249 (100)	256 (100)	251 (100)	1.000
Statin	1,004 (100)	248 (100)	249 (100)	256 (100)	251 (100)	1.000
ACEI/ARB	496 (49.40)	119 (47.98)	123 (49.40)	119 (46.48)	135 (53.78)	0.392
Beta blocker	507 (50.50)	122 (49.19)	128 (51.40)	119 (46.67)	138 (54.98)	0.288
CCB	265 (26.39)	79 (31.85)	62 (24.90)	64 (25.00)	60 (23.90)	0.161
Diuretic	141 (14.04)	34 (13.71)	36 (14.46)	41 (16.02)	30 (11.95)	0.617
Hypoglycemic agents	274 (27.29)	58 (23.39)	68 (27.31))	69 (26.95)	79 (31.47)	0.247

ApoB/ApoA-I: Apolipoprotein B/Apolipoprotein A-I ratio; BMI: body mass index; PLT: platelet; WBC: white blood cell; ALT: alanine aminotransferase; AST: aspartate aminotransferase; eGFR: estimated glomerular filtration rate; UA: uric acid; TC: total cholesterol; HDL-C: high-density lipoprotein cholesterol; LDL-C: low-density lipoprotein cholesterol; Lp (a): lipoprotein(a); TG: triglyceride; BNP: brain natriuretic peptide; DM: diabetes mellitus; AMI: acute myocardial infarction; ACEI/ARB: angiotensin-converting enzyme inhibitor/ angiotensin receptor blocker; CCB: calcium channel blocker.

### 3.2 Apob/ApoA-I ratio and the prevalence of ISR after successful DES-based PCI

**Figure 2:**
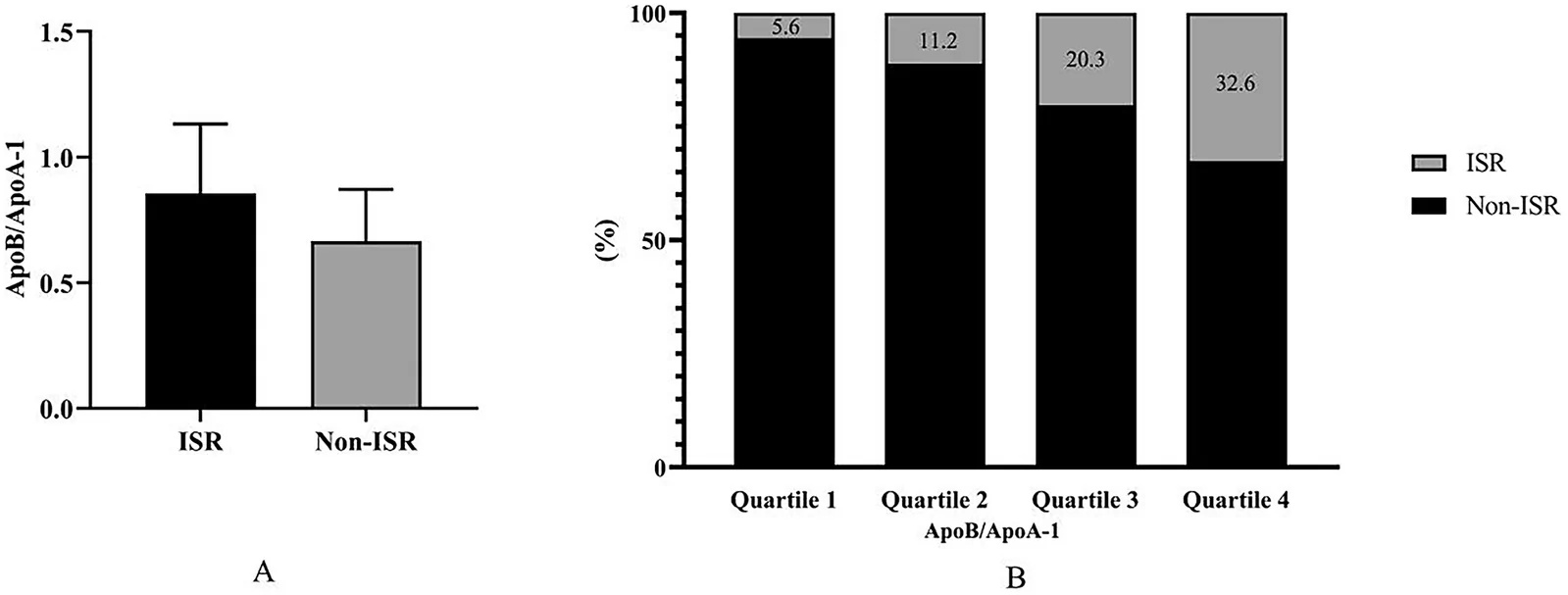
Comparison of the ApoB/ApoA-I levels between the ISR and non-ISR groups (**A**); the impact of the ApoB/ApoA-I on the incidence of ISR in the overall population **B**).

During a median follow-up period of 12 months, ISR occurred in 176 patients (17.5%). The ApoB/ApoA-I ratio was significantly higher in the ISR group than the non-ISR group (0.86 ± 0.28 vs. 0.67 ± 0.21, *P* < 0.001), as shown in Figure 2A. Furthermore, the incidence of ISR increased stepwise across ascending quartiles of the ApoB/ApoA-I ratio (5.6%, 11.2%, 20.3%, and 32.6%; *P* < 0.001), as illustrated in Figure 2B.

### 3.3 Association of the ApoB/ApoA-I ratio with the risk of ISR

**Figure 3:**
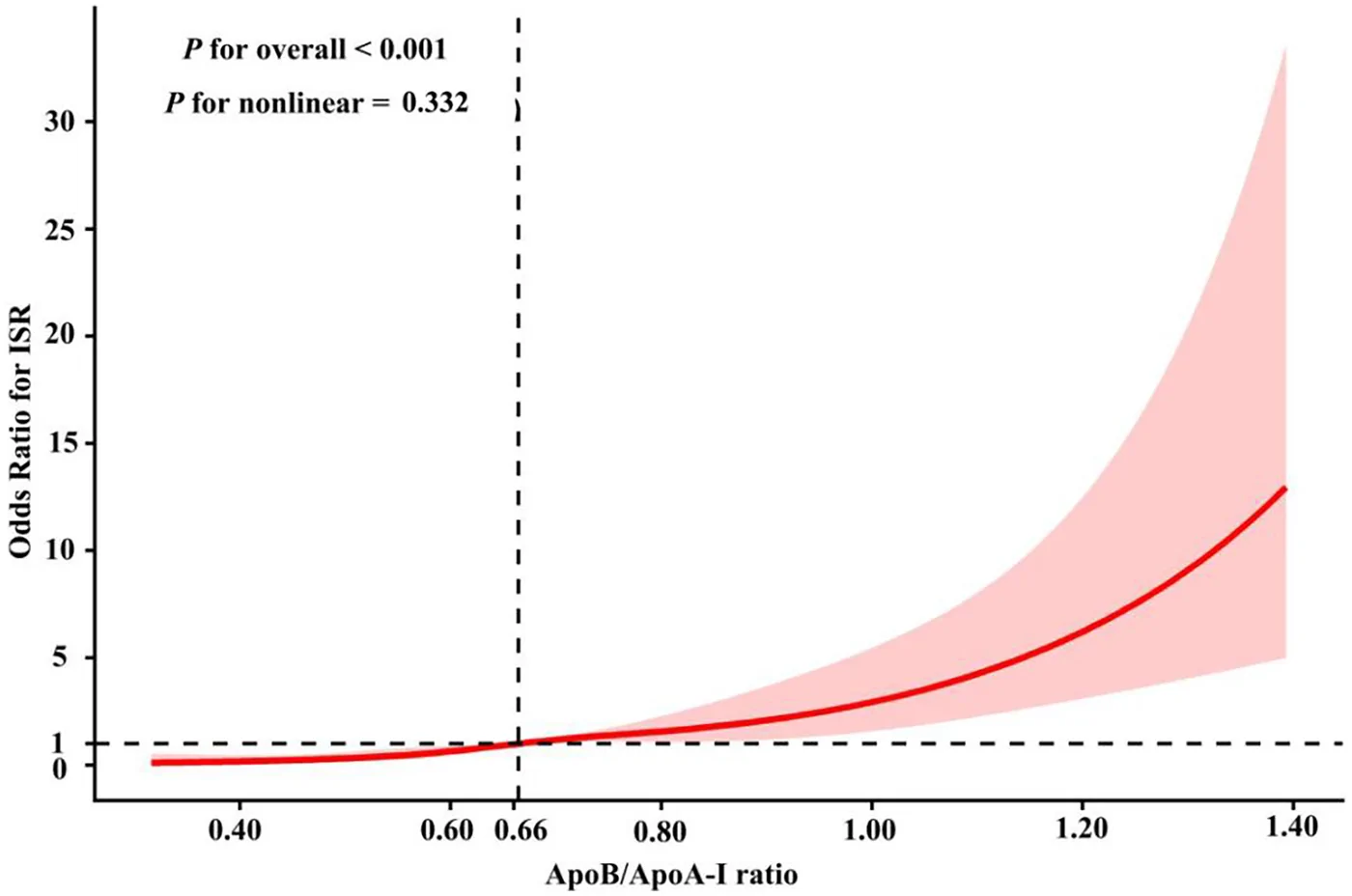
RCS plot of the association between the ApoB/ApoA-I ratio and ISR. Model was adjusted for age, male, smoking, drinking, BMI, DM, hypertension, AMI, WBC, PLT, glucose, UA, eGFR, TC, HDL-C, TG, BNP, ACEI/ARB, the number of diseased vessels, number of implanted stents, and average stent diameter.

As presented in Table 2, in the univariate logistic regression analysis (Model 1), the ApoB/ApoA-I ratio, analyzed as a continuous variable per 1-standard deviation (SD) increase, was positively associated with the risk of ISR after successful DES-based PCI (OR = 2.14, 95% CI: 1.82–2.53, *P* < 0.001). When the ratio was analyzed as categorically by quartiles, with the first quartile serving as the reference, the risk of ISR was significantly higher in the second (OR = 2.12, 95% CI: 1.09–4.13, *P* = 0.028), third (OR = 4.26, 95% CI: 2.29–7.91, *P* < 0.001), and fourth quartiles (OR = 8.11, 95% CI: 4.45–14.78, *P* < 0.001). In multivariable logistic regression analyses, with the ApoB/ApoA-I ratio examined as a continuous variable, a 1-SD increase remained independently associated with an increased risk of ISR in Model 2 (OR = 2.15, 95% CI: 1.81–2.57, *P* < 0.001), Model 3 (OR = 2.49, 95% CI: 1.90–3.26, *P* < 0.001), and the fully adjusted Model 4 (OR = 2.39, 95% CI: 1.81–3.15, *P* < 0.001). The association also persisted across all the three multivariable models when the ration was evaluated by quartiles (Table 2). After full adjustment for potential confounders in Model 4, the adjusted OR (95% CI) for patients in the third and fourth quartiles were 3.48 (95% CI: 1.67–7.22, *P* = 0.001) and 6.09 (95% CI: 2.66–13.93, *P* < 0.001), respectively, compared with those in the first quartile. Furthermore, RCS analysis confirmed a positive linear dose-response relationship between the ApoB/ApoA-I ratio and ISR risk (*P* for overall < 0.001, *P* for nonlinear = 0.332), as illustrated in Figure 3.

**Table 2:** Multivariate logistic regression analysis of the association between the ApoB/ApoA-I ratio and the risk of ISR.

Variable	OR	95% CI	*P* value
Model 1			
ApoB/ApoA-I, per 1-SD	2.14	1.82–2.53	<0.001
Quartile 1	1	Reference	—
Quartile 2	2.12	1.09–4.13	0.028
Quartile 3	4.26	2.29–7.91	<0.001
Quartile 4	8.11	4.45–14.78	<0.001
*P* for trend	—	—	<0.001
Model 2			
ApoB/ApoA-I, per 1-SD	2.15	1.81–2.57	<0.001
Quartile 1	1	Reference	–
Quartile 2	2.18	1.11–4.28	0.023
Quartile 3	4.07	2.17–7.62	<0.001
Quartile 4	7.83	4.22–14.52	<0.001
*P* for trend	—	—	<0.001
Model 3			
ApoB/ApoA-I, per 1-SD	2.49	1.90–3.26	<0.001
Quartile 1	1	Reference	–
Quartile 2	2.01	0.99–4.10	0.054
Quartile 3	3.78	1.83–7.80	<0.001
Quartile 4	6.69	2.95–15.15	<0.001
*P* for trend	—	—	<0.001
Model 4			
ApoB/ApoA-I, per 1-SD	2.39	1.81–3.15	<0.001
Quartile 1	1	Reference	—
Quartile 2	1.96	0.96–4.02	0.066
Quartile 3	3.48	1.67–7.22	0.001
Quartile 4	6.09	2.66–13.93	<0.001
*P* for trend	—	—	<0.001

Model 1 was unadjusted; Model 2 was adjusted for age, male, smoking, drinking, and BMI; Model 3 further adjusted for DM, hypertension, AMI, WBC, PLT, glucose, UA, eGFR, TC, HDL-C, TG, BNP, and ACEI/ARB based on Model 2; Model 4 additionally adjusted for the number of diseased vessels, number of implanted stents, and average stent diameter based on Model 3.

### 3.4 Subgroup and sensitivity analyses

We examined the interaction between the ApoB/ApoA-I ratio and pre-specified subgroups regarding the risk of ISR. As presented in Table 3, subgroup analyses consistently demonstrated that a higher ApoB/ApoA-I ratio, analyzed as a continuous variable (per 1-SD increase), was significantly associated with an increased risk of ISR across all subgroups (all *P* < 0.05). Moreover, no significant interaction effects were observed between the ApoB/ApoA-I ratio and any of the subgroup variables (all *P* for interaction >0.05). In a further sensitivity analysis, the ApoB/ApoA-I ratio remained significantly associated with ISR risk even after excluding 283 patients with AMI, whether treated as a continuous or a categorical variable (Table 4). Specifically, in Model 4, each 1-SD increase in the ratio yielded an OR of 2.46 (95% CI: 1.73–3.51; *P* < 0.001), while the highest quartile gave an OR of 11.29 (95% CI: 3.68–34.67; *P* < 0.001).

**Table 3:** Subgroup analysis of the ApoB/ApoA-I ratio and the risk of ISR.

Subgroup	*N* (%)	OR (95% CI)	*P* value	*P* for interaction
Gender				
Male	639 (63.65)	2.59 (1.84–3.63)	<0.001	0.201
Female	365 (36.35)	2.35 (1.35–4.09)	0.002	—
Age				
<65 years	363 (36.16)	2.06 (1.32–3.21)	0.001	0.063
≥65 years	641 (63.84)	2.88 (1.94–4.27)	<0.001	—
Smoking				
No	582 (57.97)	1.99 (1.33–2.98)	<0.001	0.782
Yes	422 (42.03)	3.24 (2.13–4.95)	<0.001	—
Drinking				
No	765 (76.20)	1.64 (1.17–2.29)	0.004	0.497
Yes	239 (23.80)	10.16 (4.63–22.28)	<0.001	—
BMI				
<24 Kg/m^2^	482 (48.01)	2.48 (1.59–3.88)	<0.001	0.856
≥24 Kg/m^2^	522 (51.99)	2.40 (1.64–3.53)	<0.001	—
eGFR				
<90 ml/min/1.73 m^2^	415 (41.33)	2.27 (1.46–3.55)	<0.001	0.724
≥90 ml/min/1.73 m^2^	589 (58.67)	2.74 (1.87–4.00)	<0.001	—
Hypertension				
No	401 (39.94)	2.36 (1.50–3.70)	<0.001	0.256
Yes	603 (60.06)	2.47 (1.71–3.56)	<0.001	—
DM				
No	710 (70.72)	2.75 (1.95–3.88)	<0.001	0.081
Yes	294 (29.28)	2.23 (1.31–3.82)	0.003	—

Model was adjusted for age, male, smoking, drinking, BMI, DM, hypertension, glucose, TC, UA, eGFR, AMI, WBC, PLT, HDL-C, TG, BNP, ACEI/ARB, number of diseased vessels, number of implanted stents, and average stent diameter.

**Table 4:** Sensitivity analysis of the ApoB/ApoA-I ratio and the risk of ISR after excluding AMI patients.

Variable	OR	95% CI	*P* value
Model 1			
ApoB/ApoA-I, per 1-SD	2.44	1.98–3.00	<0.001
Quartile 1	1	Reference	—
Quartile 2	2.62	0.99–6.92	0.052
Quartile 3	6.42	2.62–15.75	<0.001
Quartile 4	15.50	6.49–37.01	<0.001
*P* for trend	—	—	<0.001
Model 2			
ApoB/ApoA-I, per 1-SD	2.39	1.93–2.97	<0.001
Quartile 1	1	Reference	–
Quartile 2	2.62	0.99–6.93	0.053
Quartile 3	6.16	2.49–15.22	<0.001
Quartile 4	14.44	5.97–34.94	<0.001
*P* for trend	—	—	<0.001
Model 3			
ApoB/ApoA-I, per 1-SD	2.58	1.82–3.67	<0.001
Quartile 1	1	Reference	—
Quartile 2	2.77	0.82–6.25	0.114
Quartile 3	5.31	1.94–14.59	<0.001
Quartile 4	11.82	3.88–35.95	<0.001
*P* for trend	—	—	<0.001
Model 4			
ApoB/ApoA-I, per 1-SD	2.46	1.73–3.51	<0.001
Quartile 1	1	Reference	—
Quartile 2	2.11	0.79–6.15	0.129
Quartile 3	4.95	1.79–13.67	0.002
Quartile 4	11.29	3.68–34.67	<0.001
*P* for trend	—	—	<0.001

Model 1 was unadjusted; Model 2 was adjusted for age, male, smoking, drinking, and BMI; Model 3 further adjusted for DM, hypertension, WBC, PLT, glucose, UA, eGFR, TC, HDL-C, TG, BNP and ACEI/ARB based on Model 2; Model 4 additionally adjusted for the number of diseased vessels, number of implanted stents and average stent diameter based on Model 3.

### 3.5 Predictive performance and incremental effects of the ApoB/ApoA-I ratio for ISR risk

**Figure 4:**
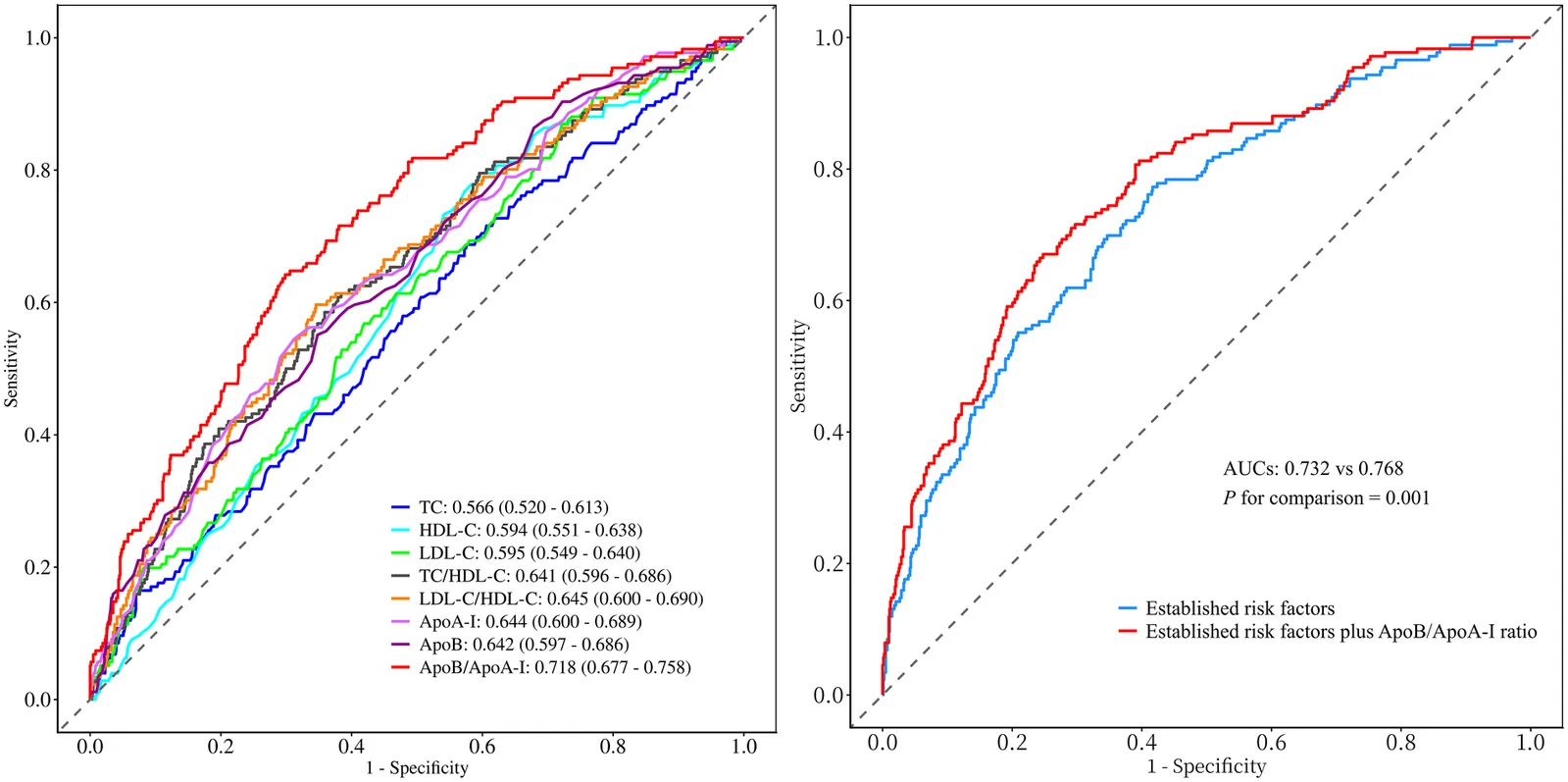
Receiver operating characteristic curve analysis of the ApoB/ApoA-I ratio and other conventional lipid-related parameters to predict DES-ISR (**A**) and comparison of the C-statistics between the models (**B**).

To evaluate the predictive performance of the ApoB/ApoA-I ratio for ISR risk, ROC curve analysis was performed (Figure 4). The AUC for the ApoB/ApoA-I ratio in predicting ISR risk was 0.718 (95% CI: 0.667–0.758). The optimal cut-off value was determined to be 0.74, yielding a sensitivity of 64.2% and a specificity of 70.1%. Compared with conventional lipid-related markers, the ApoB/ApoA-I ratio showed superior predictive performance, with the highest discrimination than TC (AUC = 0.566; 95% CI: 0.549–0.640), HDL-C (AUC = 0.594; 95% CI: 0.551–0.638), LDL-C (AUC = 0.595; 95% CI: 0.549–0.640), TC/HDL-C ratio (AUC = 0.641; 95% CI: 0.596–0.686), LDL-C/HDL-C ratio (AUC = 0.645; 95% CI: 0.600–0.690), ApoA-I (AUC = 0.644; 95% CI: 0.600–0.689), and ApoB (AUC = 0.642; 95% CI: 0.597–0.686) (Figure 4A). Meanwhile, the model of established risk factors incorporating sex, smoking, BMI, TC, HDL-C, TG, number of diseased vessels, and average stent diameter yielded a C-statistic of 0.732 (95% CI: 0.691–0.772) (Table 5). As presented in Table 5 and Figure 4B, adding the ApoB/ApoA-I ratio to the model of established risk factors could lead to an increase in C-statistics [0.732 (95% CI: 0.691–0.772) vs. 0.768 (95% CI: 0.730–0.807), *P* = 0.001], categorical NRI [0.128 (95% CI: 0.052–0.205), *P* = 0.001] and IDI [0.053 (95% CI: 0.032–0.074), *P* < 0.001].

**Table 5:** Evaluate the predictive power of models for ISR after successful DES-based PCI.

Variable	C-statistics	*P* value	*P* for comparison	Categorical NRI	*P* value	IDI	*P* value
Established risk factors	0.732 (0.691–0.772)	<0.001	Ref	–	Ref	–	Ref
Established risk factors plus ApoB/ApoA-I	0.768 (0.730–0.807)	<0.001	0.001	0.128 (0.052–0.205)	0.001	0.053 (0.032–0.074)	<0.001

Established risk factors including sex, smoking, BMI, TC, HDL-C, TG, number of diseased vessels, and average stent diameter; NRI: net reclassification improvement; IDI: integrated discrimination improvement.

## 4 Discussion

To our knowledge, this was the first study to specifically investigate the association between the ApoB/ApoA-I ratio and the risk of ISR in patients with coronary heart disease following PCI with drug-eluting stents. The main findings were as follows: (1) a higher ApoB/ApoA-I ratio was significantly associated with an increased likelihood of ISR; (2) this association remained independent after comprehensive adjustment for clinical and stent-related confounders, and was robust across sensitivity analyses and all pre-specified subgroups; (3) a positive linear dose-response relationship was observed between the ApoB/ApoA-I ratio and ISR risk; and (4) the ratio demonstrated moderate discriminative ability for identifying ISR risk (AUC = 0.718), outperforming conventional lipid-related biomarkers. (5) Incorporation of the ApoB/ApoA-I ratio into a baseline predictive model comprising established risk factors for DES-ISR significantly improved predictive performance among patients with CHD. Taken together, these results suggested that the ApoB/ApoA-I ratio might be a valuable predictor for ISR risk stratification in this clinical population.

Our findings extended the established role of the ApoB/ApoA-I ratio as a biomarker of cardiovascular risk. Substantial clinical evidence indicates that the ApoB/ApoA-I ratio is positively associated with peripheral arterial disease (25) and major adverse cardiovascular events in the general population (20). Furthermore, it has been established as an independent risk factor for poor prognosis in conditions such as coronary heart disease (26), heart failure (27) and ischemic stroke (28). However, its specific relationship with the risk of ISR in patients with coronary heart disease following DES-PCI has not been previously elucidated. The consistent, linear association we observed filled this knowledge gap. Importantly, to address the concern that acute myocardial infarction could elicit a marked systemic inflammatory and stress response that could dysregulate lipid metabolism (29, 30) and induce acute-phase variations in the ApoB/ApoA-I ratio (31), we performed a sensitivity analysis excluding AMI patients. The persistence of a significant association in this metabolically stable subgroup reinforced the reliability of the ratio as predictor of ISR.

Regarding the predictive power of the ApoB/ApoA-I ratio for cardiovascular risk, previous studies have demonstrated that this ratio outperforms conventional lipid ratios such as TC/HDL-C and LDL-C/HDL-C in predicting cardiovascular risk (18, 32). Consistent with these findings, our present study also suggested that the ApoB/ApoA-I ratio was superior to traditional lipid parameters in predicting the risk of ISR. Furthermore, our study demonstrated that incorporating the ApoB/ApoA-I ratio into a model based on established risk factors improved the ability to identify patients at risk for DES-ISR. Although its incremental predictive value for DES-ISR was limited, integrating the ApoB/ApoA-I ratio with established risk factors for risk assessment still appears to be clinically meaningful. Moreover, unlike traditional LDL-C and HDL-C measurements, which lack full standardization, the ApoB/ApoA-I ratio can be measured using internationally standardized assays for apolipoproteins (33). These attributes make it a convenient and reproducible screening tool in clinical settings. ApoB and ApoA-I can be measured alongside with standard lipid panels, and the ratio is calculated simply as ApoB divided by ApoA-I. Thus, the ApoB/ApoA-I ratio may serve as a complementary biomarker for DES-ISR risk stratification alongside traditional lipid parameters. Patients with elevated ratios can be identified as a high-risk subgroup warranting more intensive lipid-lowering therapy and closer follow-up.

The mechanisms underlying ISR are multifactorial and not yet fully elucidated. Current evidence suggests that biological/patient-related, procedural, and stent-related factors collectively contribute to ISR development (8). In the present study, we found that the ApoB/ApoA-I ratio remained significantly associated with ISR risk even after adjusting for patient-related and stent-related factors. The association between the ApoB/ApoA-I ratio and ISR risk is likely attributable to its reflection of the systemic atherogenic lipid burden. The ratio captures the balance between pro-atherogenic and anti-atherogenic lipoprotein particles (18, 34). ApoB is a key component of atherogenic lipoprotein particles (15), whereas ApoA-I is the major protein constituent of HDL particles, which exert anti-atherogenic effects (14). Therefore, an elevated ApoB/ApoA-I ratio indicates a relative predominance of pro-atherogenic over anti-atherogenic effects, which may contribute to ISR pathogenesis. This conceptual framework is supported by prior experimental and clinical evidence. For instance, experimental studies have shown that ApoA-I can modulate biological processes relevant to stent biocompatibility, including suppression of neointimal hyperplasia, reduction of in-stent thrombosis; inhibition of in-stent neoatherosclerosis, and promotion of re-endothelialization (35). In a murine stent model, systemic infusions of ApoA-I reduce in-stent neointimal hyperplasia and restenosis (36). Clinically, a retrospective case-control study of 604 patients undergoing DES implantation identifies ApoA-I as an independent protective factor against ISR after DES-based PCI (37). Moreover, a recent integrative bioinformatics analysis reports APOB as a critical predictive biomarker for ISR in patients with coronary heart disease (38). Another study of 345 Han Chinese patients confirms that ApoB is an independent risk factor for ISR after PCI, whereas ApoA-I serves as a protective factor (39). Taken together, these findings corroborate our results and suggest that an elevated ApoB/ApoA-I ratio may act as a “warning” signal that triggers pathophysiological processes involved in ISR development and progression.

Our study has significant clinical implications. The identification of the ApoB/ApoA-I ratio as an independent predictor of ISR might improve risk stratification for CHD patients undergoing DES-PCI and could offer a potential therapeutic target for preventing ISR. Given that lifestyle modifications and certain pharmacotherapies could modulate both ApoB and ApoA-I levels, interventions aimed at optimizing this ratio might help mitigate ISR risk, a hypothesis that warranted validation in future prospective trials. Several limitations of this study should be acknowledged. First, its single-center, retrospective, and observational design precluded the establishment of a causal relationship between the ApoB/ApoA-I ratio and DES-ISR. Second, the exclusion of patients without follow-up coronary angiography might introduce selection bias and limited the generalizability of our findings. Third, ISR was primarily defined by visual assessment on angiography, which was less accurate and provides fewer pathophysiological details compared with intracoronary imaging modalities such as intravascular ultrasound or optical coherence tomography. Fourth, key procedural details, such as stent generation and manufacturer, drug coating type, lesion complexity, chronic total occlusions, calcification severity, use of intravascular imaging, and post-dilation strategy, were not systematically captured in our institutional database. Information on post-discharge medication adherence and the intensity of lipid-lowering therapy was unavailable. Consequently, residual confounding arising from these unaccounted factors could not be fully ruled out. Future studies incorporating comprehensive data on the aforementioned factors were warranted to confirm our findings. Finally, the ApoB/ApoA-I ratio was measured only at baseline; data on its longitudinal changes during follow-up were limited, and thus the role of dynamic lipid profiles in the development and progression of ISR remains unclear.

## 5 Conclusions

The ApoB/ApoA-I ratio was significantly associated with the risk of ISR in coronary heart disease patients undergoing DES-PCI. A positive linear dose-response relationship was observed between the ApoB/ApoA-I ratio and ISR risk, suggesting that this ratio may serve as a valuable predictive biomarker for ISR. Further prospective cohort studies are warranted to validate these findings and to explore whether therapeutic interventions guided by the ApoB/ApoA-I ratio can effectively reduce ISR risk after PCI.

## Data Availability

The original contributions presented in the study are included in the article/Supplementary Material, further inquiries can be directed to the corresponding author.
