# Supplementary material for: The ApoB/ApoA-I ratio and the risk of in-stent restenosis following drug-eluting stent implantation in patients with coronary heart disease: a retrospective cohort study
Source: Front Cardiovasc Med. 2026 Aug 12;13:1869582. doi: 10.3389/fcvm.2026.1869582 (PMC13507672; doi:10.3389/fcvm.2026.1869582)
Supplement: Supplementary file 1 [file Datasheet1.docx]

Table S1. Multicollinearity detection

| Variable | VIF |
| --- | --- |
| Age | 1.42 |
| Male | 1.78 |
| Smoking | 1.96 |
| Drinking | 1.47 |
| BMI | 1.17 |
| Number of diseased vessels | 1.55 |
| Number of implanted stents | 1.58 |
| Average stent diameter | 1.09 |
| PLT | 1.15 |
| WBC | 1.49 |
| eGFR | 1.46 |
| UA | 1.46 |
| Glucose | 1.49 |
| ApoB /ApoA-I | 2.49 |
| TC | 2.83 |
| HDL-C | 2.80 |
| TG | 1.60 |
| BNP | 1.14 |
| DM | 1.51 |
| Hypertension | 1.27 |
| AMI | 1.42 |
| ACEI/ARB | 1.19 |

ApoB/ApoA-I: Apolipoprotein B/Apolipoprotein A-I ratio; BMI: body mass index; PLT: platelet; WBC: white blood cell; eGFR: estimated glomerular filtration rate; UA: uric acid; TC: total cholesterol; HDL-C: high-density lipoprotein cholesterol; TG: triglyceride; BNP: brain natriuretic peptide; DM: diabetes mellitus; AMI: acute myocardial infarction; ACEI/ARB: angiotensin- converting enzyme inhibitor/ angiotensin receptor blocker

Table S2 Baseline characteristics according to the ISR status

| Variable | ISR (n= 176) | Non-ISR (n=828) | *P* value |
| --- | --- | --- | --- |
| Age, years | 66.66±10.53 | 67.46±10.649 | 0.365 |
| Male, n (%) | 118 (67.04) | 521 (62.92) | 0.302 |
| Smoking, n (%) | 97 (55.11) | 325 (39.25) | <0.001 |
| Drinking, n (%) | 56 (31.81) | 183 (22.10) | 0.006 |
| BMI, kg/m^2^ | 25.24±3.50 | 24.29±3.16 | <0.001 |
| Number of diseased vessels | 2.45±0.93 | 1.96±0.86 | <0.001 |
| Number of implanted stents, /patients | 2.69±1.75 | 2.02±1.30 | <0.001 |
| Average stent diameter, mm/patients | 3.03±0.37 | 3.12±0.39 | 0.004 |
| Total stent length, mm/patients | 56.00 (32.25, 98.50) | 40.00 (24.00, 65.75) | <0.001 |
| Hemoglobin, g/L | 132.48±19.59 | 133.54±18.55 | 0.496 |
| PLT, 10^9/L | 193.17±67.91 | 181.12±66.40 | 0.030 |
| WBC, 10^9/L | 8.26±3.84 | 7.50±2.99 | 0.004 |
| Albumin, g/L | 40.70±6.49 | 41.27±4.10 | 0.136 |
| ALT, U/L | 28.00 (18.00, 42.00) | 25.00 (18.00, 38.00) | 0.256 |
| AST, U/L | 28.00 (21.00, 56.75) | 26.00 (20.00, 40.00) | 0.060 |
| Creatinine, umol/L | 78.55 (64.73, 94.58) | 72.80 (62.13, 87.00) | 0.002 |
| eGFR, ml/min/1.73m^2^ | 90.65 (70.98, 114.28) | 97.86 (79.45, 115.47) | 0.010 |
| UA, umol/L | 381.86±108.37 | 363.88±97.76 | 0.030 |
| Glucose, mmol/L | 7.05 (5.78, 9.46) | 6.78 (5.55, 8.97) | 0.084 |
| ApoA-I, g/L | 1.25±0.28 | 1.41±0.30 | <0.001 |
| ApoB, g/L | 1.02±0.23 | 0.90±0.22 | <0.001 |
| ApoB /ApoA-I | 0.86±0.28 | 0.67±0.21 | <0.001 |
| TC, mmol/L | 4.91±1.23 | 4.60±1.12 | 0.001 |
| HDL-C, mmol/L | 1.15±0.28 | 1.27±0.35 | <0.001 |
| LDL-C, mmol/L | 3.09±1.03 | 2.75±0.95 | <0.001 |
| Lp (a), mg/L | 211.50 (120.25, 450.25) | 196.00 (110.03, 363.48) | 0.144 |
| TG, mmol/L | 1.78 (1.24, 2.84) | 1.58 (1.05, 2.47) | 0.033 |
| BNP, ng/L | 72.50 (29.00, 204.25) | 53.00 (21.00, 138.00) | 0.008 |
| DM, n (%) | 69 (39.20) | 225 (27.17) | 0.001 |
| Hypertension, n (%) | 108 (61.36) | 495 (59.78) | 0.697 |
| AMI, n (%) | 60 (34.09) | 223 (26.93) | 0.055 |
| Postoperative medication |  |  |  |
| Aspirin | 176 (100) | 828 (100) | 1.000 |
| Clopidogrel/Ticagrelor | 176 (100) | 828 (100) | 1.000 |
| Statin | 176 (100) | 828 (100) | 1.000 |
| ACEI/ARB | 99 (56.25) | 397 (47.95) | 0.045 |
| Beta blocker | 91 (51.70) | 416 (50.30) | 0.735 |
| CCB | 45 (25.57) | 220 (26.57) | 0.784 |
| Diuretic | 31 (17.61) | 110 (13.29) | 0.133 |
| Hypoglycemic agents | 64 (36.36) | 210 (25.36) | 0.003 |

ApoB/ApoA-I: Apolipoprotein B/Apolipoprotein A-1 ratio; ISR: in-stent restenosis; BMI: body mass index; PLT: platelet; WBC: white blood cell; ALT: alanine aminotransferase; AST: aspartate aminotransferase; eGFR: estimated glomerular filtration rate; UA: uric acid; TC: total cholesterol; HDL-C: high-density lipoprotein cholesterol; LDL-C: low-density lipoprotein cholesterol; Lp (a): lipoprotein(a); TG: triglyceride; BNP: brain natriuretic peptide; DM: diabetes mellitus; AMI: acute myocardial infarction; ACEI/ARB: angiotensin-converting enzyme inhibitor/ angiotensin receptor blocker; CCB: calcium channel blocker.
